# The Spectrum of Spitz Melanocytic Lesions: From Morphologic Diagnosis to Molecular Classification

**DOI:** 10.3389/fonc.2022.889223

**Published:** 2022-06-07

**Authors:** Tiffany W. Cheng, Madeline C. Ahern, Alessio Giubellino

**Affiliations:** ^1^ Department of Laboratory Medicine and Pathology, University of Minnesota, Minneapolis, MN, United States; ^2^ Masonic Cancer Center, University of Minnesota, Minneapolis, MN, United States

**Keywords:** spitz, spitz nevus, atypical spitz nevus, spitzoid melanoma, spitzoid lesions

## Abstract

Spitz tumors represent a distinct subtype of melanocytic lesions with characteristic histopathologic features, some of which are overlapping with melanoma. More common in the pediatric and younger population, they can be clinically suspected by recognizing specific patterns on dermatoscopic examination, and several subtypes have been described. We now classify these lesions into benign Spitz nevi, intermediate lesions identified as “atypical Spitz tumors” (or Spitz melanocytoma) and malignant Spitz melanoma. More recently a large body of work has uncovered the molecular underpinning of Spitz tumors, including mutations in the HRAS gene and several gene fusions involving several protein kinases. Here we present an overarching view of our current knowledge and understanding of Spitz tumors, detailing clinical, histopathological and molecular features characteristic of these lesions.

## 1 Introduction

### 1.1 History and Synonyms

In 1948, Dr. Sophie Spitz detailed the clinical and histopathologic observation of 13 melanocytic lesions which she termed “juvenile melanomas”, (1) Though these lesions greatly resembled melanomas histologically, the young age of the patients and the clinical course captured the attention of Dr. Spitz, concluding that they “probably would not behave as malignant tumors.” (1) This correct observation sparked a larger interest in these lesions, marking the importance of distinguishing them from melanoma and thus avoiding overtreatment in these young patients. Later observations of these “juvenile melanomas” in adults shifted the concept of Spitz nevi beyond purely pediatric lesions (2). Subsequently, cumulative experience allowed for description of the epithelioid and spindle cell melanocyte morphology as a distinct features of these lesions, laying the ground for a wider recognition of these lesions in daily practice by pathologists globally (3).

These histologic features have also inspired these lesions’ nomenclature which has evolved over time. There is indeed a relatively long list of synonyms, such as “spindle and epithelioid cell nevus”, “spindle-cell nevus”, “epithelioid cell nevus” and “nevus of large spindle and/or epithelioid cells”. The now most commonly used name of “Spitz Nevus” (SN) was established in 1967 by the Pathology Committee of the Queensland Melanoma Project, using an eponym honoring and marking the legacy of the late Dr. Spitz, prematurely passed away a decade earlier (4).

With the better characterization of Spitz nevi as distinct melanocytic neoplasms, the first variant, the pigmented spindle cell nevus, was then recognized and described in 1975 (5). Subsequently, several more variants of SN, including the desmoplastic, pseudogranulomatous, and a variant with halo reaction were described in 1978 (6).

Given the difficulty of differentiating between benign SN and melanoma, along with multiple reports of benign SN displaying atypical features, the concept of an intermediate lesion emerged (7). Reed et al. classified these borderline lesions as “minimal deviation melanomas” (5). The terminology and designation of “Atypical Spitz Nevus”, currently in use, emerged more recently, in the 1990s (8). Around the same time, initial efforts through molecular cytogenetic analysis culminated, in 1999, with the characterization of Spitz nevi with 11p gain, by Bastian and colleagues (9).

Following the discovery of BRAF mutations in cancers, including melanoma, it was found that benign Spitz nevi do not contain actually BRAF mutations, marking an important distinction of these tumors with other melanocytic lesions (10–12). More recently, Wiesner and colleagues found that kinase fusions and translocations are commonly found across the spectrum of Spitz neoplasms (13). In-frame kinase fusions and translocations of ROS1, NTRK1, ALK, BRAF, and RET were described, and will be described later in this review (13).

In 2016, NTRK3 kinase fusions were added to the repertoire of kinase fusions found in Spitz melanoma and the effort to find additional molecular defects continue to this day, as an estimated 20-50% of Spitz tumors remain with no known genetic mutations (14, 15). Most recently, MAP3K8 translocations have been identified as an additional genetic driver (16, 17).

**Figure 1:**
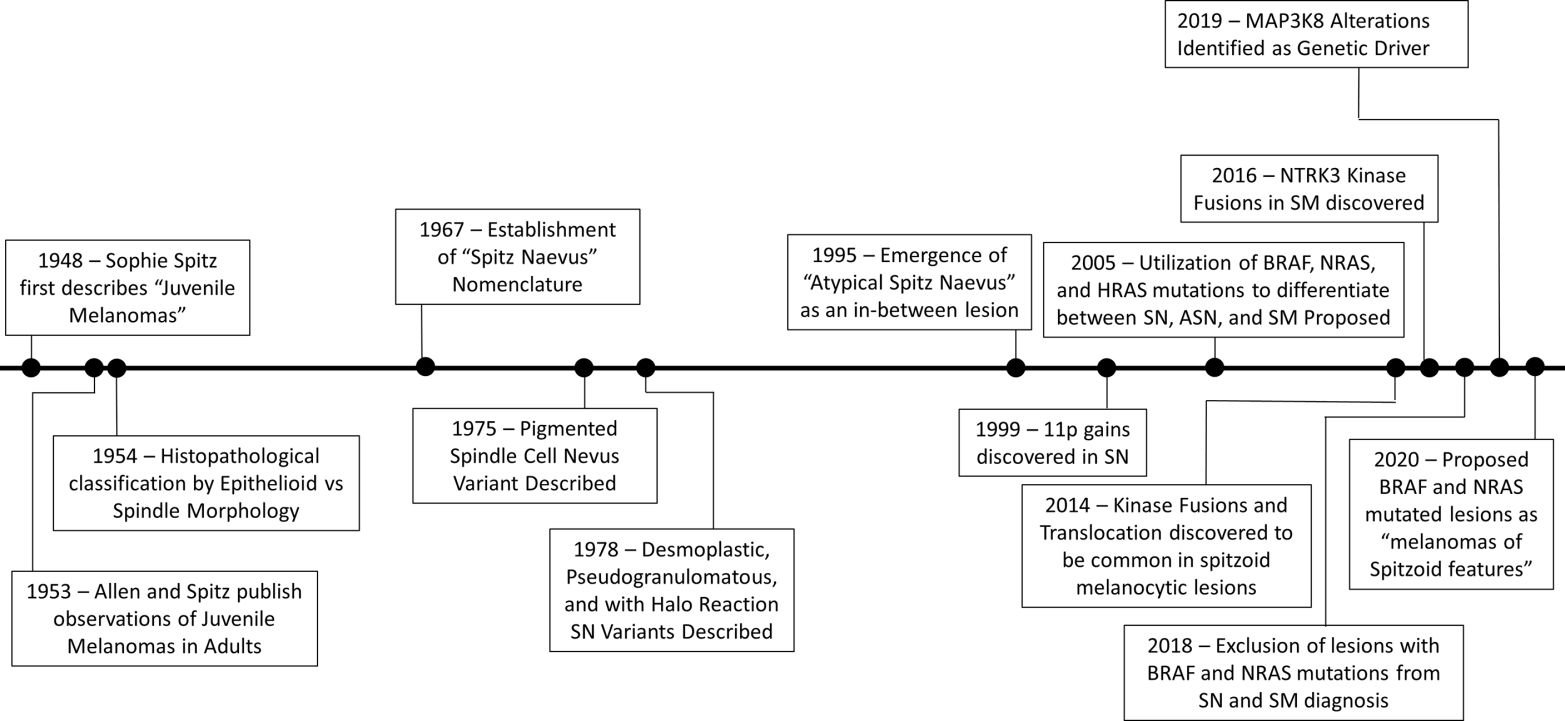
Historical Timeline of the Evolution of Our Understanding of Spitz Tumors.

A historical timeline of the evolution of our understanding of Spitz tumors is presented in **Figure 1**.

Thus, the current classification of Spitz tumor includes (a) benign Spitz nevi (SN), (b) atypical Spitz nevus/tumor (AST) as tumor of intermediate malignant potential, and (c) Spitz melanoma, a distinct subgroup of Spitzoid melanomas (SM). In defining “Spitz”, vs. “spitzoid”, the WHO 2018 guidelines define “Spitz” lesions as being associated with at least a single genetic alteration and “spitzoid” lesions as lesions with morphological resemblance to SN (18). Currently, only a little over half of spitzoid tumors are associated with a known genomic rearrangement or an HRAS mutation, and are therefore formally defined as “Spitz”, creating a problem of categorizing the remaining lesions (15). Given recent advancements in discovering new genomic aberrations, it is likely that more of these remaining spitzoid lesions will eventually be classified as “Spitz” based on a currently unknown genomic aberration.

### 1.2 Epidemiology and Pathogenesis

Detailed epidemiologic studies of Spitz tumors are difficult to perform, and few are available in the literature. First, these lesions are rare, with only 1-2% of all melanocytic lesions in all ages diagnosed as SN (18). Furthermore, even with the assistance of ancillary studies, there is generally poor concordance between dermatopathologists on the diagnosis of these lesions (19–22). However, correlations between lesion morphology with recently characterized genetic oncologic drivers can assist in identifying these lesions, facilitating accuracy of future epidemiologic studies (23).

A few estimated incidences of Spitz nevi have been reported. In 1977, Weedon and Little estimated an incidence of 1.4 cases per 100,000 individuals in Queensland, Australia (24). An incidence of 7 cases of Spitz Nevus per 100,000 individuals in Connecticut, United States has also been reported (25). More recently, an incidence of 10 cases per 100,000 individuals in the region of Emilia, Italy has been published (26). As a general estimate, 1-2% of all nevi removed from both children and adults are diagnosed as Spitz nevi (18).

The incidences of AST and SM are also unknown, though both tend to be less common than SN (18). A recent study on the yearly incidence of SN, AST, and SM in the Netherlands between 1999-2014 notably demonstrates the yearly number of SN increasing from 525 cases to 751 cases during this period; AST from 9 to 153 cases; SM from 8 to 40 cases (27). However, the general increase in incidence as well as the significant increase of AST in this study’s cases may not be due to a real increase but rather may be attributable to better recognition and clinical/histopathological/genetic classification of these lesions (27).

Currently, while the pathogenesis and etiology of SN, AST, and SM are unknown, the discovery of kinase translocations and other mutations hints to potential pathways involved in their pathogenesis. In fact, kinase fusions lead to a loss of a 3’ regulatory domain, leading to constant activation of the kinase domain portion of the fusion; this molecular event is likely responsible for cellular proliferation in these lesions (28). Described translocations include those involving *ROS1, ALK, RET, BRAF, NTRK1, MET*, and *NTRK3* (28). We will describe later, in more detail, each of these translocations.

## 2 Clinical Features

### 2.1 Demographics

Though initially termed “juvenile melanomas”, Spitz tumors occur in individuals of all ages. The mean age of diagnosis of SN in studies involving both pediatric and adult populations ranges from late-teens to early 30s, with one study reporting a mean age of 22 years (29–31). Given the later categorization of AST separate from SN and the difficult nosologic interpretation, very few studies report an average age of AST diagnosis exclusively. A mean age of 20.8 years in 55 cases of AST, has been reported, which falls within the general range of mean age of SN diagnosis (32). The mean age of SM diagnosis does tend to be higher, as expected, with a mean age of 55 years in a 54 patient study (31).

Other authors have exclusively studied pediatric populations. In a series of 512 SN and 107 AST in this younger population, the median ages for each were 7.4 and 7.2, respectively (33). Though data is limited due to the relatively rare nature of SM in a pediatric population, the mean age tends to be higher at 12.9 years (34).

SN, AST, and SM occur in all races, though the majority of cases have been reported in white patients (33, 35). SN and AST occur in both sexes; studies are split between demonstrating an equal distribution versus greater incidence in females (29–31, 36–38). Though more cases of SN and AST occurring in females are reported in the literature overall, potentially increased cosmetic attention and motivation to remove the lesion may explain, at least in part, this predominance (18, 31, 36). The WHO 2018 classification mentions Spitz Melanoma as more common in men than women; in other studies, SM appears to exhibit a relatively equal distribution between sexes, though this conclusion is limited by small sample sizes (18, 31, 35, 39).

### 2.2 Location

**Figure 2:**
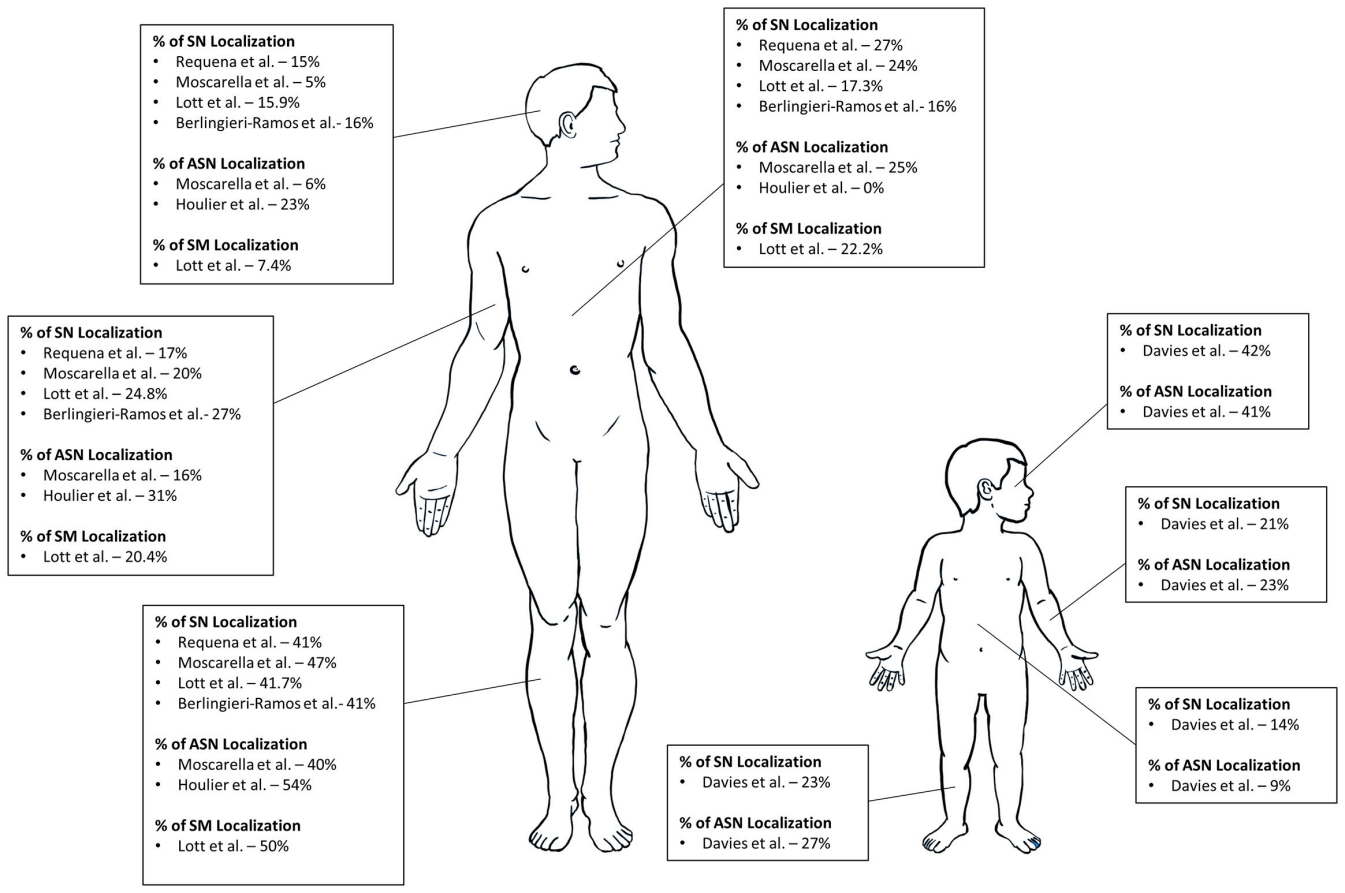
Common Localization of Spitz Tumors on Adults and Children.

SN, AST, and SM can occur anywhere in the body. The lower extremities tend to be the most common site of occurrence followed by either the trunk or upper extremities for all three types of Spitz tumors (29, 31, 36, 40). A study comparing 110 cases of SN with 55 AST showcase this localization; for SN, 47.3% were localized to the lower extremity, 20% the upper extremity, 19.9% the trunk, 4.5% the head and neck, and for AST, 40% were localized to the lower extremity, 23.6% the trunk, 16.4% the upper limbs, 5.5% the head and neck (32). A similar localization is seen in SM, with 50% located on the lower extremities, 22.2% located on the trunk, 20.4% the upper extremities, and 7.4% the head and neck (31). An illustration of Spitz tumor localization is presented in **Figure 2**.

Some studies of exclusively pediatric populations have observed higher incidences of SN and AST located in the head and neck (33, 35). In a recent study, 41.97% of 243 SN cases, 40.91% of 22 AST cases were biopsied from the head and neck (35). Due to the rarity of SM in an exclusively pediatric population, there is no consensus on where these lesions tend to manifest (33–35). Therefore, clinicians should consider potential differences in SN and AST localization in a pediatric population and remain vigilant in including SM as a differential at all sites.

### 2.3 Size, Shape, Color

Sophie Spitz described varying clinical appearances of these Spitz lesions. Most lesions were less than 1 cm. in diameter, all were elevated, and had a wide range of coloration from pink to black (1).

Conventional Spitz nevi are on average <6 mm. in diameter (18). This assertion is mostly supported in the literature; though one study does describe a mean of 76 mm (31, 38, 41). SN are classically described as dome-shaped, but may present as flat or polypoid lesions (18, 26). A pink to red color is most commonly seen in SN; flesh-colored, brown, and black lesions have also been observed (35, 37, 42).

AST are often in a range between 5 and 10 mm in diameter; a larger diameter in conjunction with appropriate histopathologic features can be a distinguishing clinical feature between AST and SN (43). Shape-wise, AST may either present as a plaque or a nodule (18). Like SN above, AST can also present in a variety of colors ranging from pink to black and like SM may be multi-colored (18).

SM are typically larger than AST, with a mean of 1.05 cm, however, SM <6mm can also occur (31, 41). SM tumors are more likely than SN to be nodular, but may also present as flat or only slightly elevated (18, 44). SM also exhibit a spectrum of color; analyzing 15 pediatric SM cases, there is a mean number of 2.7 colors per lesion, with 80% containing red/pink, 53.3% containing dark brown, 35.7% containing grey, and 33.3% containing light brown (34).

**Figure 3:**
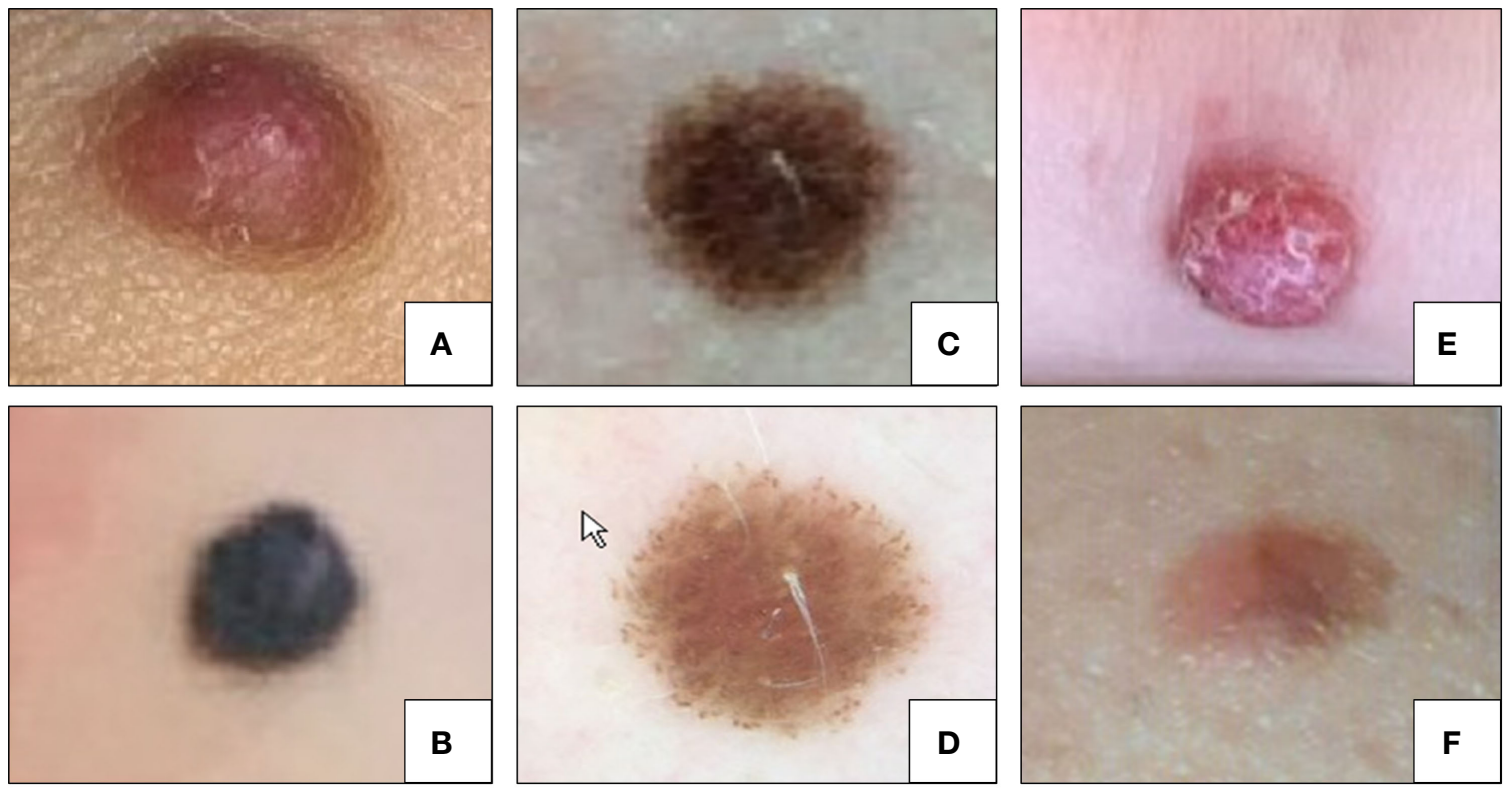
Clinical examples of Spitz nevi (histologically confirmed). **(A)** Intradermal Spitz nevus; **(B)** Pigmented spindle cell nevus of Reed; **(C)** Compound Spitz nevus; **(D)** Dermoscopy of the Spitz nevus in panel **(C). (E)** ALK translocated Spitz nevus; **(F)** “Spark” nevus.

Representative clinical pictures of Spitz lesions are illustrated in **Figure 3**.

### 2.4 Differential Diagnosis

#### 2.4.1 Clinical Diagnosis

Despite attempts to clinically characterize Spitz lesions, these lesions share many clinical characteristics, such as size, location, and color, with other skin lesions, leading to a wide differential. Spitz lesions, in particular AST and SM, can present very similarly to melanoma on physical exam, particularly with asymmetry, diameter >6mm, and color variegation along with dark brown coloration (29, 31, 34, 45). Other melanocytic lesions are also high on the differential for Spitz lesions and include congenital melanocytic nevus, atypical nevus, blue nevus, and Clark nevus (29, 31, 36, 37, 46).

Since SN commonly present with pink/red coloration, vascular lesions are also high on the differential for SN (18, 47). Common vascular lesions included in the differential for SN include hemangiomas, pyogenic granuloma, and angiofibroma (29, 36, 48). Other lesions with morphological similarity to Spitz lesions include dermatofibroma, basal cell carcinoma, and seborrheic keratoses (31, 36, 37, 46).

#### 2.4.2 Histopathologic Diagnosis

Differential diagnosis of Spitz lesions can be divided into diagnoses of melanocytic lesions and non-melanocytic lesions. Common characteristics shared between SN and other melanocytic lesions are maturation, symmetry, sharp-circumscription, and regular distribution of melanocytes individually or in nests (18, 35, 49, 50). Other lesions displaying epithelioid or spindle cell morphology are also on the differential, one example being epithelioid fibrous histocytoma which also features abundant eosinophilic cytoplasm and scattered mitotic figures (51). Some non-melanocytic lesions, such as extramammary Paget’s, melanoacanthoma, or Bowen’s disease, may demonstrate pseudo-melanocytic nests, pagetoid spread, and increased melanocytes leading to potential misdiagnosis of a Spitz lesion (52).

## 3 Dermoscopy

In 1992, Steiner and colleagues found that dermoscopy improved diagnosis of pigmented Spitz nevi from 56% to 93% (53). Since then, dermoscopic characterization has evolved and use of dermoscopy in diagnosing these lesions is now common practice among dermatologists (48). These lesions are typically classified into several dermoscopic patterns: starburst, globular, multicomponent/atypical, homogenous, reticular, and dotted vessels (32, 44, 54). The starburst pattern consists of a central blue-black pigmented area surrounded by streaks or large globules extending in a symmetric, radial manner at the periphery (32, 54). A globular pattern consists of numerous brown or grey-black round/oval structures of varying size involving most of the lesion (32, 54). A reticular pattern refers to a pigment network occupying the majority of the lesion compared to a homogenous pattern where homogenous pigmentation occupies most of the lesion (32, 54). A multicomponent pattern is where two or more of the patterns listed above are observed in a single lesion (32, 54). **Table 1** summarizes the main dermoscopic features of Spitz lesions and a dermoscopic image of a SN displaying a globular pattern evolving into a starburst pattern is shown in **Figure 3D**.

**Table 1:** Summary of Dermoscopic Features of Spitz Tumors.

SN Dermoscopic Features	AST Dermoscopic Features	SM Dermoscopic Features
• Starburst most common pattern • Nonpigmented SN associated with Dotted Vessel Pattern • Pigmented SN associated with Globular Pattern • Reed Nevus associated with Starburst pattern • Diffuse vessel distribution • Regular streaks • Regular homogeneous pigmentation • Irregular and regular brown globules • Atypical network • Blue-white veil • Inverse network	• Multicomponent or non-specific pattern more likely to be observed • Same frequency of dotted vessel as SN • White lines predictive for AST	• Similar non-pigmented SN pattern • Increased asymmetry and coloration variety • Red/pink and white coloration • Shiny white lines • Milky red areas • Polymorphous vascular patterns

The majority of Spitz nevi exhibit a starburst pattern, with one study reporting 61.3% of 333 SN (32, 44, 55). Frequencies regarding the other patterns are less consistent between studies, however, in an analysis of 15 dermoscopy studies totaling 896 SN cases, the dotted vessel pattern and globular pattern were found to be the next most frequent dermoscopic patterns seen (54). Non-pigmented Spitz nevi are commonly associated with the dotted vessel pattern, pigmented Spitz nevi with the globular pattern, and Reed nevus with the starburst pattern (56). Other dermoscopic features of SN that may be seen include diffuse vessel distribution, regular streaks, regular homogeneous pigmentation, irregular and regular brown globules, atypical network, blue-white veil, and inverse network (32).

AST are more difficult to classify given that their characteristics overlap with SN and SM. Comparing dermoscopic features of 55 AST and 110 SN, AST are more likely to exhibit a multicomponent or non-specific pattern than SN, and exhibit a dotted vessel pattern at about the same frequency as SN (32). White lines were also predictive for AST, occurring in around 20% of AST (32). Shiny white lines are also a dermoscopic attribute of SM, highlighting the difficulty of distinguishing AST from SM (34).

SM may exhibit similar dermoscopic features to SN, but in general tends to exhibit more asymmetry and more colors (34, 56). Compared to non-spitzoid melanoma, SM are less likely to exhibit a multicomponent dermoscopic pattern, atypical network, structureless areas, dark brown coloration and more likely to exhibit a pink Spitz-like pattern, red/pink and white coloration, shiny white areas, milky red areas, and polymorphous vascular patterns (34).

Excision is frequently recommended for spitzoid-like (symmetrical and asymmetrical) lesions to mitigate risk of a more atypical lesion (54). Dermoscopic monitoring is recommended in children under 12 years old. Lesions with a starburst pattern are expected to grow symmetrically in all directions with homogenous blue-black color changes before undergoing involution; lesions that do not follow this course should be excised (54). Dynamic changes in nevi are not uncommon in children and a symmetrical spitzoid lesion is unlikely to be melanoma in this population, therefore, clinicians may defer to clinical context to determine whether to pursue excision or monitoring (54, 57).

## 4 Histopathology

Although new information regarding diagnostic potential of mutations and translocations is emerging, most SN, AST, and SM are still diagnosed with histopathologic examination of hematoxylin and eosin slide in conjunction with clinical features. Findings that are typically evaluated include symmetry, circumscription, ulceration, histopathological subtype, epithelioid and/or spindle-cell nests, pagetoid spread, Kamino bodies, melanocyte maturation, mitotic activity, pigmentation, lymphocytic inflammatory infiltrate, and multinucleation (29, 30, 36, 38, 58, 59). Maturation is typically defined as the progressive decrease in cell size towards the base of the lesion (36, 59). Kamino bodies are dull pink/eosinophilic hyaline globules which are PAS and trichome positive and present in the epidermis and papillary dermis of SN (60). Histopathologic characteristics of SN, AST, and SM are summarized in **Table 2**.

**Table 2:** Histopathologic Characteristics of Spitz Tumors.

Histopathologic Feature	Spitz Nevus	Atypical Spitz Tumor	Spitzoid Melanoma
*Size*	<6 mm	6-10 mm	>1 cm
*Symmetry*	Symmetrical	May be symmetrical or asymmetrical	Asymmetrical
*Circumscription*	Well-Circumscribed	May be well or poorly circumscribed	Poorly circumscribed
*Ulceration*	Rare	More common than SN	Present
*Epithelioid/Spindle Cell Morphology*	Can be primarily epithelioid, primary spindle cell, or combination of both; well-organized into dermo-epidermal nests found in banana bunch configuration	Can be primarily epithelioid, primarily spindle cell, or combination of both; greater epithelioid predominance in AST with MAP3K8 alterations; greater nuclear plemorphism and higher nuclear:cytoplasmic ratio	Can be primarily epithelioid, primarily spindle cell, or combination of both; irregular nesting patterns; fascicular melanocyte nests in SM with ALK fusions; dermal rosette structures in SM with NRTK1 fusions; greater epithelioid predominance in SM with MAP3K8 fusions
*Pagetoid Spread*	Uncommon, typically focal if present	If present, typicaly peripheral, involves upper epidermis	Present, may be extensive
*Kamino Bodies*	Present, located at periphery of melanocyte nests	Infrequent, may be smaller in size	Rare
*Melanocyte Maturation*	Commonly Present	May be absent	Absent
*Mitotic Activity*	Low, 0-2/mm^2^	Moderate, 2-6/mm^2^	High, >6/mm^2^
*Lymphocytic Inflammatory Infiltrate*	Commonly present	May be present	May be present
*Multinucleation*	May be present, typically found in SN of primarily epithelioid origin	May be present in AST with MAP3K8 alterations	Rare
*Epidermal Hyperplasia*	Present	Rare	Rare

### 4.1 Spitz Nevi

**Figure 4:**
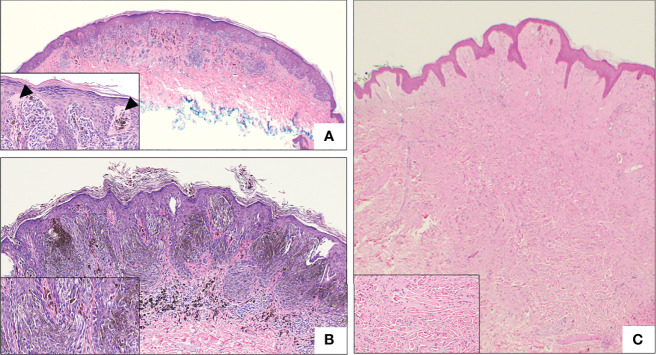
Representative histologic micrographs of Spitz nevi. **(A)** Low power overview of a compound Spitz nevus, which show symmetry and circumscription. The lesion is characterized by spindled and epithelioid cell morphology (2x). Inset: Higher power view of the same Spitz nevus with junctional nest “hanging” from the epidermis and prominent Kamino bodies (arrowhead) (20x). **(B)** Low power view of pigmented spindle cell nevus of Reed with the characteristic heavy pigmentation (10x). Inset: Higher power view of the same lesion demonstrating spindle cell morphology and heavy pigmentation (20x). **(C)** Intradermal desmoplastic Spitz nevus, showing effacement of rete ridge and an exclusively intradermal, amelanotic melanocytic proliferation. Inset: High power view showing predominantly epithelioid melanocytes distributed between thick collagen bundles.

The majority of SN are symmetrical, well-circumscribed, and pigmented (18, 30, 36, 37, 59). SN are divided into three histopathologic subtypes: compound, junctional, and intradermal, based on melanocyte location. Compound SN tends to be the most common subtype and contain both junctional or intradermal elements (**Figure 4A**) (30, 31, 36, 40). SN are synonymous with “spindle-cell and/or epithelioid cell nevus”, and as the name implies, SN are made up of large epithelioid and/or spindle melanocytes, the predominance of which varies (29, 30, 36, 38, 59). These cells are usually found in collections of nests at the dermo-epidermal junction, and are usually oriented perpendicular to the epidermis, in a configuration frequently described as “raining down” or “hanging” from the epidermis, like bunches of bananas (**Figure 4A**, Inset) (42, 50, 59). Epidermal hyperplasia is commonly observed in SN (36). Kamino bodies are often located at the periphery of melanocytic nests within the epidermis (**Figure 4A**, Inset) (50).

Although Kamino bodies are an important distinguishing diagnostic feature of SN, they are not necessarily found at the same 60% frequency Kamino et al. reported (60). In the literature, detection of Kamino bodies in SN lesions range from being found in 11% to 67% of lesions (29, 30, 36, 41). Presence of lymphocytic inflammatory infiltrate and mild to heavy pigmentation of SN are quite common (24, 30, 36, 37, 41). Maturation is also detected at high frequencies in compound and intradermal SN (30, 36, 38). Ulceration and abnormal mitotic activity are rare in SN compared to AST and SM (24, 37, 41, 61). Pagetoid spread is also relatively uncommon in SN, and when observed, is typically focal, involves the lower epidermis, located at the center of the lesion, and frequently present as cluster of cells rather than single cells (18, 30, 36, 59).

Multinucleated or mononucleated giant cells were described in the basal layer of the epidermis and superficial dermis by Dr. Spitz, and were originally considered a defining characteristic separating SN from SM (1). Despite this assertion, several studies report only finding these giant cells in <30% of lesions (29, 36, 59). However, despite the low frequency in general, two studies do also find that the majority of these giant cells were found in SN, especially the ones composed of primarily epithelioid cells (29, 36).

### 4.2 Atypical Spitz Tumor

**Figure 5:**
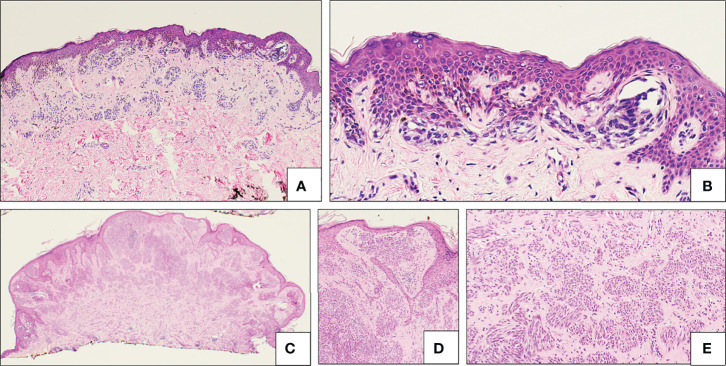
Atypical Spitz lesions. **(A, B)** “SPARK” compound nevus, showing a relatively broad atypical melanocytic proliferation **(A)**, with bridging of rete ridge and mild fibroplasia **(B)**. **(C–E)** Atypical Spitz tumor (AST), characterized by a relatively symmetrical melanocytic proliferation with little maturation with descent **(C)**, effacement of the epidermis **(D)** and relatively dense cellularity of both spindled and epithelioid melanocytes **(E)**.

Encompassing both low and high-grade tumors, AST is a more contentious diagnosis as it has characteristics of both SN and SM with uncertain malignant potential; many diagnoses are based on at least one or more abnormal histopathological findings. AST tend to be more poorly circumscribed and asymmetrical compared to SN (18, 43, 61). Comparing 50 pediatric AST cases with 20 pediatric SN cases, AST diagnosis was found to be significantly correlated with increased mitotic rate, presence of deep and atypical mitoses, nuclear pleomorphism, high nuclear/cytoplasmic ratio, absence of maturation, solid growth, and presence of asymmetry (61). Kamino bodies are even more infrequent in AST and may be smaller in size as well (43). Pagetoid spread in AST tends to be peripheral rather than focal, involves the upper epidermis, and shows distinct single-cell or small nested patterns (18). AST histology is represented in **Figures 5** **C–E**). Clonal p16 loss and heterogeneous dermal expression of HMB45 are also immunophenotypic features that AST may display (62).

There is no consensus in morphologically distinguishing AST from SM, with the designation more dependent on associated molecular pathology and retrospective diagnoses based on metastases development (18, 43). Although the ESP, EORTC, and EURACAN guidelines list morphological criterion on differentiating between a low and high grade melanocytoma, they also acknowledge AST and SM are commonly grouped into an intermediate grade and a clear cutoff of number of genetic alterations between the two has not been determined; TERT promoter alterations may help favor a malignant diagnosis (62) MAP3K8 fusions are more common in AST and SM than SN; recently, a morphologic correlation of epithelioid morphology, high grade cytologic atypia, presence of multinucleated giant cells, and p16 loss with AST harboring MAP3K8 fusions was described (17, 23). An example of AST histology is represented in **Figures 5** **C, D**).

### 4.3 Spitzoid/Spitz Melanoma

Based on the current WHO 2018 guidelines, histopathologic diagnosis of Spitz melanoma or “malignant Spitz tumours” is based on abnormal histopathological findings (18). Compared to SN, Spitz Melanoma and SM are much larger, >1 cm, and like AST, display asymmetry, poor circumscription, ulceration, irregular nesting patterns, lack of maturation, increased mitotic rate (>6mm^2^), and deep or atypical mitoses (18, 63, 64). These lesions also display high rates of cytological atypia (41, 63, 64). Despite this criterion, these rare tumors do bear a striking histopathologic resemblance to benign SN and AST, as these lesions may also display abnormal characteristics, and misdiagnoses of SM for SN/AST and vice versa based on morphology is not an uncommon occurrence (9, 19, 37).

**Figure 6:**
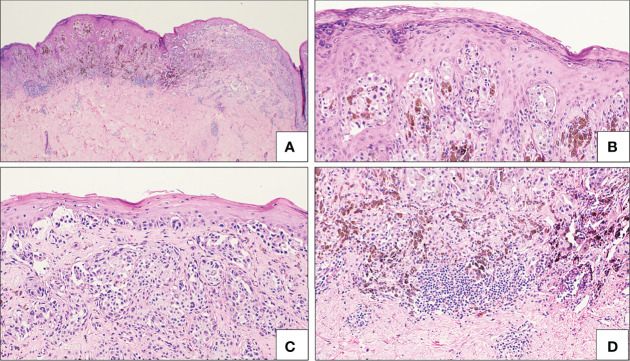
Spitzoid melanoma. There is a broad atypical compound spitzoid melanocytic proliferation with notable asymmetry **(A)**, cells in pagetoid array **(B)**, frequently single celled at the junction, alternating with irregular and confluent nests, with effacement of the epidermis **(C)**, asymmetric pigmentation and clusters of lymphocytes **(D)**. This lesion was classified as spitzoid melanoma and not Spitz melanoma based on the detection of a BRAF V600E mutation on NGS studies.

Molecular pathology is often used to confirm suspicion based on morphologic assessment. Genetic alterations associated with Spitz melanoma include kinase fusions, PTEN mutations, homozygous loss of 9p21, and HRAS mutations (18, 65). Because of the wide variance in genetic alterations in lesions morphologically categorized under SM, three subclasses of SM have been proposed: Spitz melanoma, spitzoid melanoma without identified MAPK activating mutations, and spitzoid melanoma non-spitz MAPK activating mutations (65). Spitz melanoma is defined as a morphologically diagnosed SM that also exhibits typical Spitz tumor-associated genetic alterations involving HRAS, ALK, NTRK1, BRAF, MAP3K8, and CDKN2A (65). When a non-Spitz mutation, such as a BRAF mutation, is identified, the lesion should be classified as SM; an example is illustrated in **Figure 6**.

Some Spitz melanomas retain histopathologic features of SN such as loss of p16 in those with HRAS mutations, fascicular melanocyte nests and increased dermal mitoses in those with ALK fusions, and dermal rosette-like structures in those with NRTK1 fusions (65). Spitz melanomas with MAP3K8 fusions also exhibit similar morphology to AST with MAP3K8 mutations: epithelioid morphology, high grade cytologic atypia, multinucleated giant cells, and p16 loss (17, 23). SM categorized under both spitzoid melanoma without identified MAPK activating mutations and spitzoid melanoma non-spitz MAPK activating mutations display more dermal solar elastosis and a higher number of somatic mutations (65). Further characterization of Spitz melanoma and SM and correlation of subclasses with histopathologic characteristics may play a role in more accurate future diagnoses.

## 5 Skin of Color

Due to the rarity of Spitz lesion diagnosis in non-white patients and the potential for underdiagnoses in this population, characterization of Spitz tumors in skin of color is limited to case reports and a couple of case series (46). An analysis of 130 SN cases in a Hispanic population finds that Hispanics may have a higher frequency of heavy pigmentation, appearing as a pigmented rather than pink/red papule, and a lower frequency of Kamino bodies and multinucleated giant cells (29). Another case series analyzed 11 SN and AST cases in an African-American population, finding increased prevalence in females (82%), hyperpigmentation (73%), and higher frequency of pagetoid spread (55%) compared to a white population. These reports do have limitations in the relatively small population size. However, given the importance of distinguishing benign vs. malignant Spitz tumors in combination with a general problem of misinterpretation or delayed diagnoses in patients with darker skin, clinicians should be cognizant of these potential differences, keeping Spitz lesions on their differentials (66).

## 6 Ancillary Studies

Although clinical and histopathologic characteristics remain the gold standard in diagnosing Spitz lesions, given the challenging interpretation in some lesions, ancillary studies helpful in the correct classification and prognostic interpretation. This is particularly true for borderline lesions, such as AST, where ancillary studies can contribute to assessing malignant potential.

There are a number of ancillary studies that can supplement clinical and histopathologic observations, including immunohistochemistry (IHC), array comparative genomic hybridization (aCGH), Fluorescence *in situ* hybridization (FISH), and next generation sequencing (NGS).

### 6.1 Immunohistochemistry

Immunohistochemistry (IHC) utilizes either monoclonal or polyclonal antibodies to detect specific proteins within a given tissue and is sometimes a useful tool in the diagnosis of melanocytic tumors (15, 67). IHC can detect cell cycle and/or apoptosis regulators such as Ki67, PHH3, Cyclin D1, and p16 and can be used in conjunction with melanocytic markers such as HMB45, S100/S100A6, MITF, Mart-1/MelanA, and SOX10 for assessing the general architecture of the tumor (15, 68–72).

One of the most commonly used markers, the Ki-67 protein (MIB-1), is expressed during the interphase and mitotic phases of the cell cycle (G1, G2, S, M) and is absent in the G0 phase, making it a useful marker to differentiate an actively proliferating melanoma from a quiescent melanocytic nevus (69). Compared with melanoma (where usually the ki67 proliferation index is >10% of melanocytes), SN tend to have a much lower Ki-67 proliferative index (<2% of melanocytes) and a lower dermal/epidermal count ratio (0.25 SN vs. 0.94 MM) (68, 73–75). Within Spitz tumors, SN and AST share similar expression patterns of Ki67 in the superficial dermis or at the junction, without any expression usually at the base of the lesion; AST tend to display a higher proliferative index (2-10% of melanocytes) (68). SM tends to have a high and diffuse expression throughout the entire lesion and like other melanomas tends to display a higher proliferative index (>10% of melanocytes) (68).

PHH3 (Phosphorylation of histone H3) is another helpful marker of mitotic activity that is associated with chromatin condensation in the G2 and M phases of the cell cycle (76). Although helpful in distinguishing melanoma from a benign melanocytic nevus, PHH3 does not demonstrate any advantage over and is less robust than Ki-67 (76, 77). This marker holds a similar expression pattern and proliferative index as Ki-67 for SN, AST, and SM (68). Another potentially interesting marker is Cyclin D1 which is overexpressed in more advanced lesions and may be useful as a potential differentiator of Spitz lesions; however, there are discordant and limited data in the literature (68, 78).

Loss or inactivation of CDKN2A on chromosome 9p is one of the most common molecular events in cutaneous melanoma, occurring in 50% of cases (79, 80). CDKN2A encodes the p16 protein, a cyclin-dependent kinase inhibitor that is vital to tumor suppression and whose absence promote cell cycle progression (80). SN stains strongly and diffusely for p16, but sometimes a mosaic pattern is also interpreted as a sign of benignity; on the contrary, complete absence or absence in sizable areas of the tumor, due to homozygous loss of 9p21, is an indication of loss of p16 expression and is a useful ancillary study to suspect melanoma (68, 81, 82). AST can present with either homozygous or heterozygous loss of 9p21 leading to some degree of variability in p16 positivity (80, 83).

HMB-45, Melan A, S100, MITF, SOX10, and tyrosinase are commonly used to determine melanocytic differentiation and aid in the diagnosis of melanocytic lesions (15, 84, 85). Although HMB-45 is not a particularly helpful as a lineage-specific marker, its staining pattern may help distinguish SN from SM. SN displays an organized, stratified HMB-45 staining pattern in contrast to highly variable staining frequency and distribution in melanoma, though heterogenous HMB-45 staining patterns in SN have been observed (69, 71). Commonly SN display a progressively diminished HMB-45 staining pattern; AST display either diminished or variable staining in the dermis; deep staining of the dermis is common in SM (18).

Melan-A, SOX10 and S100 tend to display identical staining patterns in melanocytic lesions, including SN (86). Subtypes of the family of S100 proteins may be of some help in distinguish different melanocytic lesions; in a study of 42 SN cases, all of these lesions strongly expressed the S100A6 protein compared with only 33% of 105 melanoma cases (72). Moreover, SN may display a diffuse pattern of S100A6 whereas melanoma appears to display a patchy distribution (21, 72). Another marker, MITF, appears to have a somewhat lower intensity in SN (18, 87, 88).

While IHC can be useful, no singular IHC stain can definitively diagnose a given Spitz lesion. Combinations of these different IHC stains may assist in algorithmically differentiating between SN, AST and SM and supplement histopathological differential diagnoses. In this context, a recent study proposes that if IHC of a lesion displays deep dermal cell proliferation through Ki67 in conjunction with complete loss of p16 and/or HMB45, the lesion is less likely a SN and more likely SM/AST; this staining pattern should prompt additional molecular investigations, such as FISH studies (80). Garola and Singh propose utilizing a combined score of p16, Ki-67, and HMB-45 (PKH score) to differentiate between SN, AST, and SM: a PKH score <4 in this study appears to be significantly associated with SN and AST, while a PKH score >4 is associated with a diagnosis of SM (70).

### 6.2 Array CGH

Array comparative genomic hybridization (aCGH) identifies copy number alterations by comparing the entire genome of a given lesion against reference DNA (15, 68). Copy number alterations are typically absent in SN, however, gains of 11p have been noted in desmoplastic SN and gains of 7q have also been reported (15, 68, 89). An AST with more benign features tend to have no more than one to two gains/losses of chromosomes, while a more atypical AST tend to have gains/losses of several chromosomes (15). No definitive single gains or losses should be used to define the malignant potential of a Spitz tumor (15). Most melanomas, including SM tend to display multiple copy number alterations (15). The limitation of aCGH is the inability to screen for other genomic aberrations, such as point mutations or balanced translocations; moreover, the input quantity of tissue can be another limitation, with false negative results when a specimen contains <50% tumor cells (15). Furthermore, aCGH is expensive, time-consuming, and labor-intensive technology, making it impractical to perform on all borderline Spitz tumors (15, 68).

### 6.3 FISH

Fluorescence *in situ* hybridization utilizes DNA probes labeled with fluorescent molecules to detect DNA sequences on metaphase chromosomes and/or interphase nuclei (90). With the identification of copy number gains and losses through aCGH, standardized FISH assay to assist in the diagnosis of melanoma have been developed and represent a viable assay to clarify the nature of problematic Spitz lesions (15, 90). The original standard melanoma FISH assay, currently still in use, test the following targets: CCND1 (11q13), RREB (6p25), MYB (6q23) and centromere 6 (15, 90). Efforts have been made to adapt this standard melanoma FISH panel to be more specific to spitzoid lesions. With the association of 9p21 homozygous deletions of CDKN2A with Spitz melanoma, a five probe assays (6p25, 8q24, 11q13, 9p21, CEN9) and a six probe assays (RREB1, CCND1, MYB, EP6, CDKN2A, and CEP9) have been developed (15, 80). FISH was found to be useful in stratifying risk in ASTs, with homozygous 9p21 deletions associated with significant aggressive behavior and intermediate risk of aggressive behavior in cases with 6p25 or 11q13 gains compared to FISH negative or 6q23 deletions (80, 91).

Though FISH is a more cost-effective measure in comparison to aCGH, FISH analysis of Spitz lesions tends to have a higher false negative rate (15, 68, 92). This is in part due to interobserver variability, thus FISH should be performed only by highly trained labs. Moreover, FISH analysis is also limited to only 4-6 genomic loci, therefore microdeletions or insertions and genomic rearrangements typically go unrecognized and/or undetected (15, 68). Therefore, a negative FISH test does not necessarily exclude the diagnosis of melanoma or, specifically a malignant spitzoid lesion. Furthermore, tetraploidy found in 5-10% of benign SN can confound FISH results with false positives for melanoma (68, 93).

### 6.4 Next Generation Sequencing

Next generation sequencing (NGS) is a powerful sequencing tool that allows parallel sequencing of millions of small DNA fragments at a time and mapping of these sequenced fragments against the human reference genome allows detection of several abnormalities (94). Compared with aCGH and FISH, NGS assays are able to assess a wider variety of genomic aberrations including point mutations, microdeletions/insertions, genomic arrangements, and copy number alterations (15). From assessing only a selected panel of genes to the entire genome, NGS is easily scalable, making it an appealing option for assisting in the diagnosis of Spitz lesions. As the cost of NGS will decrease over time, it may become a more viable alternative to other molecular studies.

In a pilot study of a new NGS-based test for melanocytic tumors, 16 benign nevi (including 7 spitzoid melanocytic lesions, 4 “epithelioid” nevi, 5 blue nevi variants) were compared against 15 melanomas (95). NGS findings in SN included BRAF, MET, NTRK1, and ROS fusions and for SM included EML4 fusion, BRAF, MAP2K1, and TERT mutations (95). Comparing FISH results against NGS for one SN and 3 SM cases, NGS concurred with positive FISH tests for 2 SM cases, showed malignancy in one FISH negative SM case, and was benign in one FISH positive SN case (95). In the same study, NGS was also able to correctly diagnose all malignant lesions (95). Thus, NGS may be able to mitigate the problem of false negatives, as we sometime see in FISH studies, and it may help to clarify the malignant potential of a melanocytic spitzoid lesion.

NGS was recently used to reclassify SN and SM from melanomas of Spitzoid features (MSF) after the WHO’s classification of skin tumors proposed elimination of lesions with *BRAF* and *NRAS* mutations from the categories of SN and SM (96). Out of 80 SN and 26 SM, 12 were reclassified as MSF based on the presence of *BRAF* and *NRAS* mutations detected by NGS and 81% of SN and 76% of SM post-reclassification displayed chimeric fusions and/or truncations (96). The majority of MSFs contained *RAF, NRAS, NF1*, and *TERT* promoter mutations and were associated with worse recurrence-free survival and metastatic potential than SN and SM (96). With the incorporation of genomic data being highly predictive of recurrence and the reclassification of lesions previously diagnosed as SN or SM through NGS, there is utility in using NGS to clarify diagnoses of SN and SM from MSF, with important implication for patient management (96). As mentioned above, though costs are declining, NGS is still significantly more expensive than aCGH and/or FISH studies and thus is not yet widely available (15).

Overall, as we learn about additional molecular determinants of Spitz lesions, the use of all these molecular studies, in conjunction with standardized protocols, will become increasingly relevant in the correct interpretation and classification of SN, AST, and SM.

## 7 Genomics of Spitz Tumors

### 7.1 Mutations in Spitz Tumors

As described above, as techniques such as comparative genomic hybridization (CGH) and fluorescence *in situ* hybridization (FISH) have become commonplace, a paradigm shift has occurred in the evaluation of Spitz lesions (30). The advent of NGS has allowed for the sequencing of a growing number of Spitz lesions, uncovering relevant mutational profiles (**Table 3**).

**Table 3:** Genomic Characteristics of Spitz Tumors.

	Type (Mutation or Translocation)	Fusion Partners	Histopathological Features	Clinical Features	Incidence
HRAS	Mutation	N/A	Low cellularity, desmoplasia, infiltrating base (97, 98)	Pleiotropic (97)	5-22% (30, 99, 100)
BRAF	Translocation	NRF1, SOX6 (65), EMLR4, BAIAP2L1 (101)	Epithelioid morphology, high-grade nuclear atypia (102)	Favors extremities, non-metastatic (102)	5% (65, 100, 102)
ROS1	Translocation	TPM3, PPFIBP1, MYH9, CAPRINI1, MYO5A (104)	Epithelioid and spindle cells homogeneously (104)	Pink/red papules in various locations (104)	7-19% (100, 104)
NTRK1	Translocation	LMNA, TP53 (105)	Superficial, evenly distributed pigmentation (106)	Favors extremities, may be amelanotic, even sex distribution, median age 26y (100)	8-19% (13, 100, 106)
ALK	Translocation	NPM1, TPR, CLIP1, GTF3C2 (107)	Fascicular growth pattern, infiltrating base, and fibrillar cytoplasm (107)	Favors extremities, may be amelanotic or vascular, more common in females, median age 12y (106, 107)	8-26% (13, 100, 106, 108)
MET	Translocation	TRIM4, ZKSCAN1, DCTN1 (13, 105)	Fusiform or epithelioid morphology, amphophilic cytoplasm, melanocyte nests (105)	Epidermal hyperplasia, good treatment outcomes (105)	0.5-1% (100, 105)
RET	Translocation	GOLGA5, KIF5B (105)	Not well described	Driver of lung cancer formation, can be treated with crizotinib, cabozantinib, and vandetanib (13)	2-3% (13, 100, 109)
MAP3K8	Both	SVIL, DIP2C, UBL3, STX7, SPECC1, CUBN, PRKACB (16)	Epithelioid morphology, high grade cytological atypia, multinucleated giant cells, p16 loss, junctional nests, desmoplasia (16, 23, 107)	Ulceration, dome-shaped, median age: 18, tendency for lymph node involvement (16, 17, 23)	8-33% (23, 28, 110)

N/A, Not Applicable.

#### 7.1.1 HRAS

“HRAS,” a proto-oncogene located on chromosome 11p, is the first genetic aberration that was associated with Spitz nevi (11, 111, 112). It is mutated (most often exons 2 or 3) in up to 15-20% of solitary Spitz nevi, but this number rises significantly in the presence of a chromosome 11p amplification (67%) (97, 99, 113). Mutations are widely varied but include 61Gln to Leu, 61Gln to Arg, and 12Gly to Arg (113). Additional genomic aberrations have been discovered and are relatively common, however, the significance of these aberrations is unknown (111). HRAS mutations have been shown on multiple occasions to aid in distinguishing Spitz nevus from Spitz melanoma, as HRAS is almost never present in melanoma (103). In a sample of over 150 melanomas, only one was found to have an amplification of HRAS (113). Because of this distinction, and due to a low rate of proliferation in these lesions, HRAS mutation is synonymous with a good clinical outcome (113). One exception to this is a study which found that amplified regions of acral melanomas (which have a poor prognosis) often contain HRAS mutations (114).

Lesions carrying this mutation show histopathological location within the dermis (though an infiltrating base may be present), low cellularity, and desmoplasia (97, 98). Generally, 11p amplifications are associated with vesicular nuclei, nuclear pleomorphism, and ample eosinophilic cytoplasm though these traits may also be present in normal 11p configurations (113). While general morphological features exist, variations are also possible. One study identified atypical morphology in HRAS mutated SN including a polyploid silhouette and a bulbous growth pattern (111). Clinically, HRAS exhibits pleiotropy, as other benign melanocytic lesions like sebaceous nevi and nevi spili often show HRAS mutations (98). In addition, these lesions are commonly found on the head and neck, with the most common secondary location being the extremities (111). On exam, the lesions are most often raised or papillomatous and most commonly present with a “wedge-shaped” silhouette. Diagnostically, when tissue material is scarce, FISH presents an attractive method of identifying a SN with an 11p gain (111).

Overall, HRAS has been found to be a selection force for 11p increasing copy numbers that is a distinguishing feature of SN that most often precludes the diagnosis of melanoma, though not always (114).

### 7.2 Translocation Spitz Tumors

#### 7.2.1 Common Translocations

##### 7.2.1.1 ROS1

**Figure 7:**
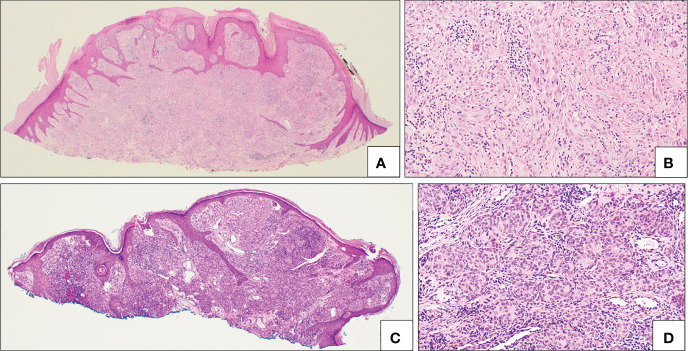
Translocated Spitz nevi. **(A, B)** Example of ALK fused Spitz nevus with a polypoid silhouette **(A)** and plexiform intersecting fascicles of fusiform melanocytes **(B)**. **(C, D)** Example of ROS fused Spitz nevus with a diffuse proliferation of melanocytes **(C)** mostly in small nests **(D)**.

ROS1 translocations are the most common, with up to 17% of SN or AST carrying this molecular finding; SM and Spitz Melanoma usually do not have this translocation (13, 104). This gene, which encodes a receptor tyrosine kinase, can have several partner genes involved in its translocation, including PWWP2A, TPM3, PPFIBP1, MYH9, CAPRINI1, MYO5A, and GOPC (110, 115, 116). While histology shows variable features, the majority of ROS1 fusions show epithelioid and spindle cells homogeneously as well as melanocytes in whorling nests and mucinous changes (104) (**Figures 7C, D**). In the largest study to date on ROS1 mutated SN, wide variability present within the microscopic appearances of these lesions was noted, therefore, many studies have claimed that identification of ROS1 mutated lesions on morphology and histology alone would be imprudent (104, 117). FISH, NGS, and immunohistochemistry are useful ancillary studies to distinguish these lesions (118). Recently, a “concomitant” cutaneous and nodal ROS1 translocated Spitz lesions has been described, with the nodal lesion displaying a biologically “indolent” pattern of metastatic spread (119).

ROS1-translocated lesions are found throughout the body, but the lower extremities appear to be the most reported. In one study of seventeen cases of ROS1 fusions, all patients were disease free at 30-months post-excision with no recurrence or metastasis (104). In cases of unresectable Spitz tumor with GOPC-ROS1 fusion, treatment with crizotinib, a tyrosine kinase inhibitor, has been shown to be effective in flattening lesions in a 20-week course of treatment (116). Interestingly, the same translocation was found in an acral lentiginous melanoma that dramatically responded to entrectinib. One smaller study (n=6) also found a greater prevalence of ROS1 mutations in females (5:1) and in younger individuals under age 40 (117). This finding was confirmed on a larger study, where 80% of ROS1 lesions were found in females, with a median age of 29 years (118). A trend towards younger age in translocated nevi has also been observed (15). ROS1 fusions are not limited to SN and AST, but may be found in acral lentiginous melanoma, as well as other cancers such as pediatric gliomas and lung adenocarcinoma, among others (115). Patients usually have an excellent prognosis. More recently, a study of 11 cases of ROS1-rearranged lesion, predominantly AST with nested pattern, has confirmed the above findings, and identified four patterns of ROS1 immunostaining, but with no correlation with specific fusion partners (118).

##### 7.2.1.2 NTRK1-3

Gene fusion in NTRK1, 2 and 3 have been reported in a range of Spitz tumors. NTRK fusions were first reported in Spitz tumors by Wiesner et al, reporting gene rearrangement in NTRK1 in 16% of the 140 spitzoid lesions analyzed, including SN, AST and SM (13). In their paper the reported fusion partners were LMNA (most common) and TP53. NTRK1, a gene encoding for TRKA, is a particularly attractive target for cancer therapy, owing to its status as a critical protein in carcinogenesis (100). Activation of NTRK1 has been shown to play a role in cell signaling pathways such as PI3K/AKT and MAPK, affecting cell survival, proliferation, and regulated cell death (13). Morphologic analysis of NTRK1 fused Spitz neoplasms has revealed a small spindled cell morphology with Kamino bodies and frequent rosette formation. More recently, a filigree-like architectural pattern of the rete ridges has been described (100), as well as rosette-like features of dermal melanocytes, in Spitz tumors with NTRK1 fusion. A novel NTRK2-TFG gene fusion was reported last year in a nevus of Reed, identified through a screening with a pan-TRK immunohistochemistry antibody and confirmed through RNA sequencing (120).

NTRK3 kinase fusions were reported by Yeh and colleagues with fusion partners including ETV6, MYO5A and MYH9 (14). Interestingly, NTRK3 fusion appears to be the most typical genomic aberration in pigmented spindle cell nevus of Reed, a variant of Spitz nevus which is highly pigmented and clinically worrisome (121). In this series, 57% of Reed nevi carried a NTRK3 translocation, with similar partner genes previously reported (14). In a recent large series of 180 ASTs, a screening pan-TRK immunohistochemistry assay revealed 26 positive cases; of these, NTRK1 fusion were detected in 15 cases and NTRK3 in one case, confirmed by various techniques, including FISH, RNA-based NGS and RT-PCR (122).

##### 7.2.1.3 ALK

The anaplastic lymphoma kinase (ALK) genes encode for a receptor tyrosine kinase in the insulin receptor family. First discovered in anaplastic large cell lymphoma (ALCL) as a driver translocation, ALK gene alterations have been described in several different tumors, including melanocytic neoplasms. ALK immunohistochemistry is a reliable and cost-effective marker that can be used to screen for potential translocation in this gene.

In the original series from Wiesner and colleagues, ALK translocated spitzoid neoplasms represented 10% of SN, about 15% of AST and 3% of SM, with DCTN1 and TPM3 as the 5’ fusion partners, rearrangements validate by FISH (13). A series of 17 spitzoid neoplasms with ALK fusion, reported from the same group, has shown a classic clinical presentation as polypoid nodule and a predilection of these lesions for the lower extremities (40). A clinical image of an ALK translocated SN can be found in **Figure 3E**. Histologically they present as a compound lesion with a predominant intradermal melanocytic component of plexiform intersecting fascicles of fusiform melanocytes (**Figures 7A, B**). Another series of 32 ALK translocated Spitz tumors, has shown novel fusion partners (CLIP1, GTF3C2) in additions to the ones already reported in other tumors (TPR, NPM1) (107). Clinically these lesions present predominantly in the extremities as exophytic papules and histologically were composed of elongated nests of spindled melanocytes, in a wedge-shaped/bulbous distribution and infiltrative pattern at the periphery. No Kamino bodies are usually present. An additional fusion partner, MLPH (melanophilin), involved in melanosome trafficking, has been recently described in pediatric Spitz nevi (123). More recently additional partners were found in an investigation of 29 cases of ALK-translocated Spitz tumors, including KANK1, MYO5A and EEF2 (124).

Interestingly, most lesions are amelanotic and are the largest lesions among all the translocated Spitz neoplasms. They also present more commonly in the extremities of young patients. Overall, it appears that the category which is most represented in ALK fused lesion is AST, with a minor component represented by SN and SM.

A recent large study of 352 Spitz lesions, confirmed that the distinct histologic morphology of ALK-fused Spitz tumors makes these lesions distinguishable from other translocated tumors; thus, attentive examination of routine H&E tissue section is sufficient to arise suspicion that can be confirmed by immunohistochemistry and/or additional molecular testing (23).

#### 7.2.2 Less Common Translocations

##### 7.2.2.1 BRAF

As mentioned above, BRAF mutations exclude a diagnosis of Spitz tumor, but these lesions can have a translocation in this gene as the driver oncogenic event. BRAF is found in only around 5% of spitz neoplasms, most of which appears to fall into the Spitz Melanoma category at least in one series (65). In the series of 49 spitzoid neoplasms, 14 lesions were found to have a BRAF fusion, with an average patient age of 24 years, and 50% of these lesions occurring in the extremities. In this series, most of the lesions were AST; morphologically, tumor cells were arranged in sheets, with epithelioid morphology, severe nuclear atypia, and sclerotic stroma. These findings were confirmed in other studies, where the most common fusion partner was CLIP1, in addition to SKAP2, AGK, MYO5A, MLANA and others (102).

##### 7.2.2.2 MET

The MET gene region is located on chromosome 7q and plays a central role in cell growth and motility, as well as melanocyte homeostasis. Spitz tumors carrying this translocation do not have many distinctive features to help suspect and confirm attribution to this category. However more frequently spindled and epithelioid melanocytes with amphophilic cytoplasm are present.

Both AST and SM are represented in this class.

##### 7.2.2.3 RET

RET, or “receptor tyrosine kinase REarranged during Translocation,” has been described in thyroid and other malignancies; in melanocytic pathology it comprises only about 2-3% of SN (13, 109). Little is known about its histopathological features and translocation partners in the context of SN, also due to the relative rarity of these translocations; GOLGA5 has been identified as one partner (13).

Due to the infrequency of both RET and MET translocations, it is difficult to estimate correctly the percentage of lesions with this fusion within the three categories of SN, AST or SM. Proposed treatment for RET translocated lesions includes vandetanib or cabozantinib, which are selective RET inhibitors (13).

##### 7.2.2.4 MAP3K8

Alterations of MAP3K8 (COT, TPL2), a serine-threonine protein kinase activating MEK, through removal of its final exon through fusion or truncation have recently been shown to be oncogenic drivers of Spitz tumors (16, 17, 28, 125). SVIL is the most common 3’ fusion partner found in these lesions, with other recurrent partners including DIP2C, UBL3, STX7, SPECC1, PRKACB, and CUBN (16, 17). Although identified as a target, the frequency of this mutation is still under investigation with one retrospective analysis of spitzoid tumor samples finding that 33% of SM harbor a MAP3K8 fusion and two other cohorts finding only 8% of total Spitz lesions harbor a MAP3K8 fusion (16, 23, 110). MAP3K8-rearranged cases display epithelioid morphology featuring monomorphic and amelanotic melanocytes, marked cytologic atypia, ulceration, loss of p16, and are more prevalent in AST and Spitz melanoma compared to other kinase fusions (17, 23, 28, 126). AST and Spitz melanoma presenting with lymph node metastases tend to present with MAP3K8 overexpression, indicating that presence of MAP3K8 fusions may play a role in determining prognosis of a given Spitz lesion (23, 28, 126).

## 8 Variants of Spitz Nevi

### 8.1 Polypoid

Polypoid SN is a rare variant of SN that displays concerning features resembling polypoid melanoma (127–129). These lesions are pedunculated with large epithelioid or spindled melanocytes with prominent atypia including large nuclei and prominent nucleoli (127–129). Unlike polypoid melanoma, polypoid SN are typically monomorphic, negative for aberrant chromosome copy numbers, and mitotic figures are much more difficult to detect (127–129). Polypoid SN also demonstrate a mesenchymal component with increased stromal collagen, similar to the desmoplastic variant of SN and other benign melanocytic lesions (127, 129). A case of polypoid AST demonstrating asymmetry and moderate nuclear polymorphism was reported recently that demonstrated a homozygous deletion of 9p21 and a *CLIP2-BRAF* fusion (130).

### 8.2 Agminated

Agminated SN are eruptions of multiple (>2) conventional SN in a disseminated or clustered “agminate” pattern, though cases of a dermatomal distribution have been reported (131–135). Three different categories have been described: “Grouped on grossly normal skin,” “Grouped on hypopigmented macule,” and “Grouped on congenital hyperpigmented macule.” (135) The disseminated pattern is rare and is more commonly found in adults; the agminated pattern, in particular agminated arising on hyperpigmented skin, is more common and is associated with children (133, 134).

### 8.3 Pagetoid

The Pagetoid SN was first described by Busam and Barnhill in 1995 and is characterized by a prominent pagetoid distribution of epithelioid melanocytes typically located in the spinous cell layer of the lesion (136, 137). The melanocytes are individually distributed (136, 138). Melanoderma, mild dermal inflammation, epidermal hyperplasia, eosinophilic bodies can be seen in Pagetoid SN (138). Clinically, these lesions are small (diameter <6mm), symmetrical, usually located on an extremity, and are well-circumscribed (136–138). Young children are associated with Pagetoid SN, however, one case series found an average age of 34 years; due to the rarity of this variant, further studies are needed to clarify this demographic (136–138).

Superficial spreading melanoma *in situ* is a common differential for these lesions (137). However, the distribution of melanocytes in superficial spreading melanoma *in situ* is less orderly and confined than Pagetoid SN and are clinically larger and asymmetrical (137). FISH positivity for 6p25, 6q23, Cep6, and 11q13 may be helpful in diagnosis of superficial spreading melanoma *in situ* over Pagetoid SN (139).

### 8.4 Dysplastic/SPARK

Dysplastic/SPARK SN are a variant of SN that displays characteristics of both classic SN and Clark’s nevi (dysplastic nevi) (140, 141). In 1978, Clark et al. found that moles that exhibited “B-K mole syndrome”, now known as “dysplastic nevi” displayed atypical melanocytic hyperplasia, lymphocytic infiltration, and angiogenesis, increasing the risk of melanoma (140, 142). Analyzing 2164 compound melanocytic nevi, 67 (3.1%) lesions demonstrated epidermal and dermal features of SN (141). A clinical image of Spark SN can be found in **Figure 3F**.

Histopathologically, either spindle and/or epithelioid melanocyte nests are located in both a hyperplastic epidermis and dermis in an architectural pattern reminiscent of Clark’s nevi, including dermal fibroplasia, denser lymphocytic infiltrate, and cytologic atypia (140, 141) (**Figures 5A, B**). The junctional component in compound lesions displays lateral extension and the spindle cells, if present, are typically parallel to the epidermis (140, 141). Kamino bodies may also be found in Dysplastic/SPARK SN (140). Clinically, Dysplastic/Spark SN are small (<1 cm), symmetric, well-circumscribed and display a female predominance (140). Based on a study of 27 cases, mean age at diagnosis is 33 years old, with a range of 6-64 years; these lesions do not recur or metastasize (140).

### 8.5 Desmoplastic

Initially, the prominent stromal sclerosis, decreased cellularity, and increased deposition of collagen characteristic of Desmoplastic Spitz Nevus were thought to be age-related change in the natural history of the SN (5, 143, 144). However, better characterization of these lesions have led to the contestation of its classification as either a subtype of Spitz tumor or as a separate entity (5, 6, 144–146).

Histopathologically, Desmoplastic SN are often intradermal or compound with little junctional activity and composed of either large spindle and/or epithelioid melanocytes arranged in small nests or solitarily distributed between thick collagen bundles (6, 137, 144). The melanocytes display a variety of phenotypes including type A/B nevus cells, ovoid melanocytes, dendritic melanocytes, and Spitzoid melanocytes (144). (**Figure 4C**). Many of these lesions are amelanotic though variable levels of melanin have been observed and mitotic figures are uncommon (137, 145, 146).

Clinically, Desmoplastic SN are asymptomatic lesions that may present on the head and neck or the extremities (137, 144, 145). They average 3.5 mm in diameter and are typically symmetric, wedge-shaped, and well-circumscribed (144, 147). Because of the striking fibrosis and sparse melanin found in these lesions, they are often confused for fibromas and desmoplastic melanoma (137).

Although desmoplastic melanoma is high on the differential, there are several distinguishing characteristics. Desmoplastic melanomas are typically larger, asymmetrical without sharp circumscription, and located in sun-exposed areas (137, 147). Desmoplastic melanomas have deeper involvement, with the melanocytic nests located deeper than those found in Desmoplastic SN (137, 145). The melanocytes in Desmoplastic melanomas display a fibroblastic-like appearance with hyperchromic nuclei (145). Small lymphocyte aggregates in the dermis are found in both Desmoplastic SN and Desmoplastic melanoma, however, these are typically accompanied with dermal mitotic figures in Desmoplastic melanoma (137, 147). HRAS expression and/or low Ki-67 positivity on immunohistochemistry or isolated gain of 11p make a diagnosis of Desmoplastic SN more likely (137, 147).

### 8.6 Hyalinized/Hyalinizing

Hyalinized SN is a variant of SN and may even be considered a variant of the Desmoplastic SN (137). Extensive and prominent stromal hyalinization distinguish hyalinized SN as its own entity (148, 149). These lesions may be composed of spindle or epithelioid melanocytes that may be arranged in nests, a single-file arrangement, or as solitary cells in the setting of their characteristic hyalinized stroma (148, 149). Clinically, these lesions are small, symmetric, tan-pink, raised, and can be found anywhere on the body (148, 149).

### 8.7 Angiomatoid/Angiomatous

Angiomatoid Spitz Nevus is a rare benign variant of SN with less than 10 cases reported (150, 151). These lesions are intradermal with no epidermal involvement and are characterized by stromal fibrosis, sparse proliferation of spitzoid melanocytes, and have abundant small, thick-walled blood vessels (137, 150, 151). Because of the numerous blood vessels present in this lesion, it may be confused for an angioma; additionally, the hypervascular stroma may imply a diagnosis of regressed melanoma (137).

Because of shared clinical and histopathologic features, Angiomatoid Spitz Nevus may be considered a subtype of Desmoplastic SN (137, 150). The presence of solitary melanocytes rather than nests, stromal fibrosis, low cellular density, and asymptomatic clinical presentation are common between both types of lesions (150, 151). High vascularity and lack of spindle cells with hyperchromatic nuclei distinguish Angiomatoid SN from Desmoplastic SN (150).

### 8.8 Combined

Although combined nevi are commonly associated with blue nevi, combined SN is a relatively rare variant of combined nevi displaying populations of spitzoid melanocytes combined with one or more other distinct nevomelanocytes (152–154). Typically, Combined SN are composed of either large epithelioid or spindle spitzoid melanocytes combined with either banal nevus, dysplastic nevus, or blue nevus cells (152–154). Clinically, combined SN occur in patients of all ages and display a female predominance (152, 153). These lesions are small, symmetrical, and well-circumscribed (152).

Histopathologically, the spitzoid melanocytes are typically located more superficially and are accompanied by other characteristics of SN including epidermal hyperplasia, clefts between nests of melanocytes, and lymphocytic infiltrates (152, 153). The other nevomelanocytic populations may be located laterally to the spitzoid melanocytes, above the spitzoid melanocytes, or may be intermingled with spitzoid melanocytes throughout the lesion (152). Though combined SN is uncommon, more atypia, including cytologic atypia, mitotic activity, and deep dermal infiltration accompanied with increased cellularity are found in these lesions than in other common types of combined nevi (153). Dermal sclerosis may also be observed (153).

With this combination of relatively high rates of atypia and dermal sclerosis, invasive melanoma is high on the differential for combined SN (152, 153). Careful identification of deep dermal mitosis is important in differentiating between combined SN and melanoma (153). Melanoma will typically be larger, asymmetrical, and display poor circumscription (152).

### 8.9 Myxoid

Myxoid Spitz Nevus is an exceedingly rare variant of SN, with fewer than 10 cases published in the literature (36, 155–157). First described in 1990, this variant presents with a myxoid stroma and mucin deposits may be present in the stroma between either isolated or nests of melanocytes (155–157). Clinically, these lesions are small, symmetrical, and may be compound or junctional (157). One study detected Kamino bodies in four out of six cases of Myxoid SN (157). These lesions bear a strong resemblance to malignant myxoid melanomas and care must be taken to rule out malignancy (157, 158).

### 8.10 With Halo Reaction

SN with Halo Reaction is a rare variant of SN. Generally, a “halo reaction” is a finding of dense lymphocytic infiltrates throughout a part of or the entirety of a lesion histologically but may lack a clinical halo of depigmentation visible to the naked eye (159). A dermal inflammatory reaction consisting of either lymphocytes or eosinophils or plasma cells infiltrating the symmetrically distributed nests of epithelioid and/or spindled melanocytes is characteristic of SN with Halo Reaction (6, 159, 160). The halo reaction in SN with Halo Reaction may occur in conventional SN or in combined SN; though there may be a slight predominance of halo reaction in combined SN (159–161).

In conventional SN with halo reaction, characteristics of SN are preserved including epidermal hyperplasia, Kamino bodies, and clefts between melanocytes and keratinocytes (159). The “halo reaction” is symmetrically distributed throughout the whole thickness of the lesion, stopping at the dermal-epidermal junction (159). These lesions are pigmented, symmetrical, sharply circumscribed, wedge-shaped, and commonly do not present with clinical halos although the possibility does remain (159–161). Few mitotic figures are observed (159).

In compound SN with halo reaction, a conventional SN phenotype was commonly seen combined with common compound or intradermal nevus or with superficial congenital-type nevi (159, 160). In general, the “halo response” is confined to the SN component of the lesions, with the lymphocytes symmetrically distributed within the SN component (159). Deep mitotic figures are absent in these lesions (159, 161). Although compound SN with halo reaction may appear asymmetrical, raising concern for melanoma, a distinguishing characteristic in compound SN with halo reaction is that each distinct nevomelanocytic component should appear symmetrical when analyzed separately (159).

### 8.11 Pigmented Epithelioid

Pigmented Epithelioid SN (PESN)are a variant of SN characterized by a heavily pigmented lesion composed entirely of epithelioid cells; it may also be considered the epithelioid counterpart to the Pigmented Spindle Cell Nevus of Reed (162, 163). The PESN is much rarer than the PSCN, with fewer than 10 cases in the literature (162, 163). Striking epidermal hyperplasia is characteristic of these lesions along with nests of epithelioid melanocytes present at the dermo-epidermal junction (162, 163). Morphologically, PESN appears similar to the epithelioid blue nevus; however, blue nevi do not typically feature a prominent junctional component (163).

Additionally, the PESN must be distinguished from the Pigmented Epithelioid Melanocytoma (PEM); both lesions present with abundant heavily pigmented epithelioid cells with junctional nests (163–165). No specific genetic alterations have been associated with PESN yet; PEM is associated with fusions in PRKCA and inactivation of PRKAR1A (164, 165). Complicating the matter, PRKAR1A-inactiated PEM have recently been found to harbor genomic anomalies associated with other melanocytic tumors, including Spitz tumors, therefore, PRKAR1A inactivation may be a secondary genetic event in a broad group of nevi, including Spitz nevi (165). Hence, the question of whether the PESN is its own entity or some variant of PEM represents an area for further investigation.

### 8.12 Tubular

Tubular SN is a rare variant of SN featuring prominent tubular or microcystic structures in the setting of aggregates of cuboidal epithelioid cells (166). These lesions do not have mitotic figures, cellular atypia, or inflammation (166). Tubular SN stains positive for the S-100 and NKI/C3 melanocytic markers on immunohistochemical analysis (166). Clinically, tubular SN resembles SN as a small, pigmented or pink, symmetric lesion (166). Although accepted as a variant of SN, there is conjecture that the “tubular” structures observed in this lesion are merely an artifact of formalin fixation, and therefore, tubular SN should not be considered a separate variant (167).

### 8.13 Pseudogranulomatous

Pseudogranulomatous SN is characterized by a dense lymphocytic infiltrate surrounding nests of epithelial melanocytes located at the dermal-epidermal junction (6, 36, 168). The epidermal hyperplasia characteristic of SN is often accompanied with collarette (6, 168). Pyogenic granuloma is high on the differential for these lesions and clinically, they present as red, soft, sessile papules in young patients (168). Pseudogranulomatous SN stain positive for S-100 and HMB-45 and CD45 and CD3 can be detected in the lymphocytic infiltrate (168).

### 8.14 Recurrent/Persistent

Recurrence is rare in SN, however it is known that melanocytic nevi may recur post-excision (169). The reported mean time to recurrence is 13-17 months and the average age reported is 14.7 years (169, 170). Stern’s analysis of 16 recurrent SN cases found that recurrent lesions were intradermal, featured prominent fibrotic stroma, and the clusters of melanocytes extended deeper into the deep reticular dermis and/or the subcutaneous fat (170). Four general types of recurrent SN have been characterized: a pseudomelanoma-like lesion with intraepidermal involvement, a typical SN lesion arising on a scar, a melanoma-like lesion with nodular growth pattern, and a desmoplastic SN-like lesion (169). Greater cytologic atypia, deeper mitoses, asymmetry, pagetoid spread, and lack of maturation may also be observed in recurrent SN (169). Clinically, these lesions may be larger than the original SN and extend beyond the biopsy scar; they may be either nodular or elevated (169). Immunohistochemical staining of recurrent SN for Ki-67 and HMB-45 may display identical reactivity patterns to SN (low proliferation Ki-67 and strong dermal HMB-45 staining), and may be helpful in distinguishing between recurrent SN and melanoma (169).

### 8.15 Reed Nevus/Pigmented Cell Nevus

The Reed Nevus or pigmented spindle cell nevus (PSCN) was first described by Reed et al. in 1975 (5). Its designation as a separate entity vs. a SN variant remains controversial, but is currently considered by the 2018 WHO Classification to be a “distinct variant of Spitz naevus.” (18) Like SN, PSCN are small (mean 3 mm), benign lesions that primarily occur on the lower extremities of young adults (mean 25 years), with a predilection for women (18) (171–173). A clinical image of PSCN can be found in **Figure 3B**. PSCN are well-circumscribed and symmetrical papules or nodules with dark brown, bluish-black, or black coloration (**Figure 4B**) (18, 171). Unlike SN which can consist of primarily epithelioid cells, PSCN are characterized by spindle-shaped, uniform, finely pigmented cells that are either vertically oriented, whorled, or concentric fascicles (**Figure 4B**, inset) (18, 171, 173). Epidermal hyperplasia is also common (171, 173). Pigmented Kamino bodies may be observed in the lower epidermis and were found in 12% of lesions (18, 171).

In addition to symmetry and good circumscription, compared with superficial spreading melanoma, PSCN have a low mitotic count, less lymphocytic inflammation nest uniformity, more epidermal hyperplasia and pigmentation, and moderate/marked presence of melanophages (174). As mentioned above, PSCN tend to exhibit a dermoscopic starburst pattern, that may later evolve and stabilize into a homogenous or reticular pattern (56, 175). A superficial black network, with a reticular appearance, due to increased melanin in these lesions is another dermoscopic feature common in PSCN (56). In a recent analysis of 23 PSCN, 57% of PSCN exhibited NTRK3 fusions compared to 3% of SN, AST, and SM combined (121). Complete excision of these lesions as treatment is recommended due to potential risk of melanoma misdiagnosis (171).

Like PSCN, atypical PSCN exhibit characteristic fascicles of uniform, spindle-shaped pigmented cells (176). However, they may exhibit asymmetry, poor circumscription, increased lymphocytic inflammation, ulceration, melanocytic hyperplasia spreading peripherally along the basal layer of the epidermis, abnormal mitotic activity, and other cytological atypia (18, 176). Higher numbers of epithelioid cells may also be seen in atypical PSCN (176).

### 8.16 Plexiform Spindle Cell Naevus

The plexiform spindle cell naevus (PLXSCN) is a variation of the PSCN with a distinct wedge-shaped plexiform architecture of pigmented spindle cell fascicles found in the reticular dermis differentiating them from other spitzoid lesions (176–178). Reported mean age is 33.1 years, with a relatively equal gender distribution and are also most commonly found on the extremities (177). These lesions are slightly elevated and tend to be blue, blue-brown, or blue-black, often being mistaken for blue nevi or melanoma (177, 178). These lesions average 3mm and tend to also be symmetrical, well-circumscribed with low mitotic activity and mild cytologic atypia (177). Atypical PLXSCN exhibit more cytological atypia including greater mitotic rate, increased nodular confluence, and larger size (177).

## 9 Prognostic and Predictive Features

SN is a benign diagnosis with a favorable outcome; if excised, these lesions do not tend to recur (30, 37, 179, 180). Seven cases of recurrence in SN excised with positive margins have been reported, making positive margins a potential predictor of recurrence (35). One occurrence of SN developing into distant metastases leading to death has been described, highlighting the difficulty of clinically and histopathologically differentiating SN from SM/Spitz melanoma or melanoma (48). This difficulty is further highlighted in a recent study where two lesions determined to be benign SN by the majority of expert dermatopathologists, actually developed distant metastases (19). Overall prognosis largely depends on accurately identifying SN.

The malignant potential of any given AST remains difficult to predict as many ultimately benign AST exhibit abnormal features (19). A comparison of 13 dermatopathologist opinions on the malignant potential of 75 AST found low agreement in determining lesion malignancy (19). In general, a very small percentage of AST have been reported to develop into metastases. Only 0.7% of 1237 AST diagnosed in the Netherlands were reported to have developed metastases; a separate study reports one out of 76 AST cases as evolving into a metastatic lesion (27, 179). Older age, lesion size >1cm, ulceration, subcutaneous fat involvement, and mitotic activity >6mm^2^ are factors that can contribute to the malignant potential of AST (181).

A homozygous 9p21 deletion may predict an AST’s malignant potential. Comparing outcomes of 16 AST with heterozygous 9p21 deletions with 22 AST with homozygous 9p21 deletions, no heterozygous 9p21 AST developed local-regional disease or distant metastases whereas 22% of the homozygous 9p21 AST developed local-regional disease, 18% developed metastases, and 9% died from disease (83). The presence of TERT promoter mutations may also predict negative outcomes in AST (182). AST harboring MAP3K8 kinase rearrangements may be more prone to local lymph node metastases and regional evolution (23, 126). The utility of other mutations or translocations to predict malignant potential warrants further investigation.

SM has a poor prognosis compared to AST and SN (41). Prognosis of SM compared with melanoma is unclear, complicated by the evolving genomic definition of SM leading to underdiagnosis of SM (18). One study found no significant differences in mortality or metastases development between SM and melanoma while others report a lower mortality rate for SM compared to MM; these conclusions are all limited by small sample sizes (183–185). Older age, expectedly, appears to correspond with negative outcomes; the 5-year survival rate for children <10 is 88% and for children 11-17 years old, 49% (186). Similar to AST harboring MAP3K8 kinase rearrangements, SM with this translocation may present with advanced disease than those without (23, 126).. Given the nascent distinction of Spitz melanoma from SM, further work needs to be done to determine prognosis between these two types of melanomas, and it is possible that current Spitz melanoma fatalities may be attributed to spitzoid melanoma or vice versa.

## 10 Management

Currently, no consensus guidelines exist for the management of SN, AST, and SM. Given the resemblance to melanoma, the majority of dermatologists still choose to biopsy a suspected SN, especially in older patients (187, 188). Excision is typically recommended to avoid recurrence, and most dermatologists will typically re-excise a lesion with positive margins (48, 187, 188). Excision of SN that have undergone shave biopsies with negative margins is unnecessary, as negative margins imply the lesion has been completely removed, and observation is sufficient (180). If clinicians do elect to observe a lesion rather than excise it, follow-up intervals of 15 days for nodular lesions found in adults and children and intervals of 2-3 months for flat/raised lesions in adults or until dermoscopic stabilization in children are recommended, as discussed earlier (54). Given the benign nature of these lesions, sentinel lymph node biopsy (SLNB) is not necessary or recommended for management.

Due to the unknown malignant potential of any given AST, excision of the lesion is recommended and in practice, re-excision of the lesion post-biopsy, regardless of margin status, is common (180, 188). The role of SLNB is controversial, as AST by definition should not metastasize to lymph nodes, and thus should not require SNLB. Studies of morphologically diagnosed AST have found AST with positive SNLB and have found no difference in metastatic potential or outcome in cases with or without positive lymph nodes (189, 190). Given that many of these lesions were diagnosed through histopathologic evaluation rather than genomic profile, it is possible that these AST with lymph node metastases may represent a type of SM instead. Thus, SNLB is not indicated for genomically-defined AST.

Currently, SM is typically managed using the same guidelines as conventional melanoma (191). With the advent of a defined Spitz melanoma as a molecularly separate entity from conventional melanoma and the low incidence of recurrence/relapse associated with SM, treatment of SM/Spitz melanoma may not require the same aggressive treatment protocol currently utilized in melanoma management (191). Overall, further molecular studies differentiating SM and Spitz melanoma from melanoma as well as further insight into the pathophysiology and prognosis of SM/Spitz melanoma are required in order to develop management guidelines.

## 11 Other Considerations

### 11.1 BIMT (BAP1-Inactivated Melanocytic Tumor)

The discovery that inactivating mutations of BAP1 (BRCA1 associated protein-1) lead to a familial predisposition for development of melanocytic tumors resembling AST sparked investigation of the loss of BAP1 in Spitz lesions (192, 193). This subset of AST featured loss of BAP1, often in conjunction with BRAFV600E mutations, and spitzoid morphology including epithelioid melanocytes in sheet-like growth with large vesicular nuclei (193, 194). However, many histopathologic characteristics of SN are not found in these lesions including epidermal hyperplasia, junctional supranest clefting, spindled melanocytes, and Kamino bodies (193, 194). As mentioned earlier, BRAF mutations are not considered a distinct hallmark of Spitz tumors. Therefore, since the molecular profile of BIMT is markedly distinct from those of conventional SN and can be associated with other common nevi, BIMT is now considered a separate entity and no more considered as a Spitz lesion (18, 194, 195).

### 11.2 Other Spitzoid Lesions

Some melanomas may display a small component of spitzoid features including granular pigmented or glassy eosinophilic cytoplasm (137). Other MMs may arise in or be associated with a conventional SN (137). As both types of tumors typically exhibit the same behavior as melanoma, defining these lesions as SM is not recommended (137).

Nevoid melanoma refers to melanoma displaying morphology of conventional or congenital benign melanocytic lesions (137). These lesions may display some overlapping morphologic features with SM such as epidermal hyperplasia (137). However, *NRAS* and *BRAF* activating mutations are characteristic of these lesions which rules out a diagnosis of SM (137).

Currently, melanocytic lesions featuring MAP2K1 mutations are being studied to determine whether categorization as “Spitz” is appropriate (196, 197). These lesions range from benign to malignant and exhibit a diversity of phenotypes (196). Melanocytic Neoplasms with MAP2K1 lesions exhibit some spitzoid cytomorphology including large melanocytes featuring vesicular nuclei; some lesions also feature a prominent desmoplastic stroma as seen in Desmoplastic SN (196, 197). However, cases reported in the literature are few and other genetic features of these lesions, for example, lack of HRAS mutation or co-existing BRAF mutation, may argue against a diagnosis of a Spitz lesion (196, 197). Therefore, despite spitzoid features, it is currently difficult to definitively correlate a morphological diagnosis of a Spitz tumor with a MAP2K1 mutation.

Three cases of RASGRF1-rearranged spitzoid neoplasms have recently been identified (198). All three lesions displayed spitzoid morphology including nests of spitzoid epithelial/spindled melanocytes and epidermal hyperplasia; fusion partners included: CD63, EHBP1, and ABCC2 (198). Given that RASGRF1 is a guanine nucleotide exchange factor gene rather than a kinase-encoding gene, although these lesions are spitzoid in morphology, it is still too early to determine whether a RASGRF1 genomic aberration classifies this spitzoid lesion as a Spitz lesion (198).

## 12 Discussion/Conclusion

Since Dr. Sophie Spitz’s initial description of “juvenile melanomas” in 1948, there has been a dramatic increase in the body of knowledge surrounding Spitz tumors. Although diagnosis of these lesions still heavily relies on clinical and histopathologic characteristics, the discovery of mutations and translocations associated with these lesions is reshaping the field, improving identification of these lesions. Decreased costs of useful ancillary studies such as NGS will improve accessibility to these tests and assist in the genetic diagnosis of Spitz tumors. Moving forward, these associations between morphology and genetic will help in better classification and will better inform the clinician not only in the potential behavior and prognosis of the lesion, but also, in case of a malignant diagnosis, in the identification of specific molecular targets for therapy.

Interest in the utilization of artificial intelligence (AI) and machine learning to aid in clinical diagnoses of various medical conditions has exponentially grown in the past 20 years. Availability of improved training and advanced pattern-recognition algorithms and development of computer hardware capable of high-speed processing make the implementation of AI into the clinical setting more feasible (199). Within the field of dermatology and dermatopathology, applications of AI and machine learning include diagnosis of various lesions *via* AI recognition of clinical, dermatoscopic, and histopathology images (200–202). Comparison of AI-diagnosed skin cancers with clinicians assessments has been shown to be comparable to the diagnostic skills of board-certified dermatologists (200, 203).

Investigation into applying AI to the identification of Spitz lesions has already begun, with one convolutional neural network demonstrating sensitivity, specificity, and accuracy of 85%, 99%, and 92%, respectively, in differentiating whole slide images of SN from conventional nevi (204). As discussed earlier, genetic subtypes of SM are correlated with different histopathologic characteristics and potential differences in prognosis (17, 23, 65). Therefore, the future implementation of AI and machine learning to correlate histopathology or even dermatoscopic images to newly identified subtypes of Spitz lesions may increase accurate and timely detection of these lesions, particularly important in the recognition of SM/Spitz melanoma.

One limitation to the development of AI algorithms, as well as clinician identification of suspicion lesions, is the limited availability of numerous, high-quality images of these lesions. In order to improve both AI and clinician identification of Spitz lesions, efforts must be made to compile a diverse, well-represented database of clinical, dermatoscopic, and histopathology images of SN, AST, and SM/Spitz melanoma. Furthermore, particular care should be taken to include images of Spitz tumors in skin of color given different clinical and histopathologic presentation (29, 46). Incorporation of these images into textbooks and educational materials will be vital to improving medical education and clinician identification of Spitz lesions.

Overall, we present a holistic view on the spectrum of Spitz melanocytic lesions, synthesizing currently known clinical, histopathological, and molecular classification of SN, AST, and SM/Spitz melanoma. Given the recency of new molecular classifications of Spitz lesions, and in anticipation of new discoveries, further long-term studies on prognosis of these different subtypes are required to develop management guidelines. Better identification of these lesions through expansion of medical knowledge and the incorporation of new technologies such as AI will facilitate early identification and treatment of malignant lesions such as SM as well as avoidance of unnecessary biopsies or re-excisions in benign SN.

## Author Contributions

TC, MA, and AG all contributed to the conception, writing, and editing of this paper, including design of figures and tables. All authors contributed to the article and approved the submitted version.

## Conflict of Interest

The authors declare that the research was conducted in the absence of any commercial or financial relationships that could be construed as a potential conflict of interest.

## Publisher’s Note

All claims expressed in this article are solely those of the authors and do not necessarily represent those of their affiliated organizations, or those of the publisher, the editors and the reviewers. Any product that may be evaluated in this article, or claim that may be made by its manufacturer, is not guaranteed or endorsed by the publisher.
